# Trends in childhood pneumococcal vaccine coverage in Shanghai, China, 2005–2011: a retrospective cohort study

**DOI:** 10.1186/s12889-016-2785-7

**Published:** 2016-02-02

**Authors:** Matthew L. Boulton, Nithin S. Ravi, Xiaodong Sun, Zhuoying Huang, Abram L. Wagner

**Affiliations:** 1 University of Michigan School of Public Health, 1415 Washington Heights, Ann Arbor, 48109 MI USA; 2 University of Michigan Medical School, Ann Arbor, MI 48109 USA; 3 Centers for Disease Control and Prevention, Shanghai, China

**Keywords:** China, Migrants, Pneumococcal vaccination, Vaccine co-administration

## Abstract

**Background:**

In China, the pneumococcal conjugate vaccine (PCV7) and the pneumococcal polysaccharide vaccine (PPSV23) are not offered under the government’s Expanded Program on Immunization and are instead administered for a fee. PCV7 is more effective and covers more serotypes associated with invasive disease in children, but is also more expensive, than PPSV23. Because of their expense, there is concern that these vaccines, especially PCV7, have low uptake particularly among non-locals, migrants from outside of Shanghai. This paper characterizes the differential coverage of PCV7 and PPSV23 between locals and non-locals in Shanghai, and illustrates coverage trends over time.

**Methods:**

In this retrospective cohort study, children born between 2005 and 2011 were sampled from the Shanghai Immunization Program Information System. Bivariate and multivariable analyses examined the relationships between demographic characteristics, residency status (non-locals vs locals), and vaccination coverage.

**Results:**

PPSV23 coverage (29.8 %) among children over 2 years of age was higher than PCV7 coverage (10.1 %) for locals and non-locals. Uptake of PCV7 increased substantially after children were 2 years of age. Overall, non-local populations had higher PPSV23 coverage (OR: 1.34; 98 % CI: 1.22, 1.46) but lower PCV7 coverage (OR: 0.617, 98 % CI: 0.547, 0.695) than locals.

**Conclusions:**

There is a need for increasing overall pneumococcal coverage in Shanghai children, particularly with the more effective PCV7 vaccine. Morbidity and mortality due to invasive pneumococcal disease for children <1 year of age are unlikely to be mitigated if the current age-related vaccination patterns are not improved.

## Background

The pathogen *Streptococcus pneumoniae* (pneumococcus) is the most common cause of pneumonia in children worldwide [1], and it is the etiologic agent for invasive pneumococcal disease (IPD), comprising meningitis, bacteremia, and pneumonia [1]. Globally, there are 14.5 million cases and 826,000 deaths each year due to IPD in children younger than 5 years. Twelve percent of these cases occur in China, and approximately 30,000 children <5 years die from IPD there each year [2].

Pneumococcal infections are largely preventable through routine immunization. There are ninety different pneumococcal serotypes capable of causing infection in humans, and the two pneumococcal vaccines currently available in China cover a number of the most prevalent strains that cause IPD. The 23-valent pneumococcal polysaccharide vaccine (PPSV23) has been available in China since 1996 and has several drawbacks: it only elicits short-term immunity, cannot be administered until 2 years of age, and has a lower efficacy ranging from 60 to 70 % at preventing IPD [3, 4]. The 7-valent pneumococcal conjugate vaccine (PCV7) was introduced to China in 2008, although it was pulled off the market in 2015 because its import license expired [5]. It protects against fewer serotypes, but has better efficacy (>90 %) [4], and it can be administered earlier, at 6 weeks of age [4]. This moves up the timeline of protection and represents a major advantage over the PPSV23 since the incidence rate of IPD is highest in children <2 years of age [4].

PCV7 has had a marked impact on reducing the burden of IPD in countries that have made it widely accessible to children through inclusion in their recommended immunization schedule. In the US, which formally recommended PCV7 for use in 2000, IPD caused by the serotypes in the vaccine decreased by 94 % (from 15.5 to 1.0 cases per 100,000 population) between 1998 and 2007 [6]. In contrast, neither the conjugated or polysaccharide pneumococcal formulation of the vaccine has yet been included in the Chinese Expanded Program on Immunizations (EPI), unlike many other countries around the world [7]. China’s reluctance to include either pneumococcal vaccine in its EPI is due to a combination of factors, including the high cost of vaccine, the lack of serotype research on the most prevalent strains in China, continuing questions regarding the actual burden of IPD in Chinese children, and concerns over vaccine efficacy [8].

Similar to in other countries, vaccines on the Chinese EPI, such as diphtheria-tetanus-pertussis vaccine (DTP), oral poliomyelitis vaccine, and the measles-containing vaccines, are fully and publically funded, are mandatory for school entry, and have higher uptake by the general public than vaccines not included on the EPI schedule. Parents are required to pay out of pocket for their children’s non-EPI vaccines, including the *Haemophilus influenzae* type b vaccine (Hib), rotavirus vaccine, and varicella vaccine [9]. The PPSV23 vaccine is produced domestically in China and costs approximately 23 US dollars whereas the PCV7 vaccine is imported and costs approximately 130 US dollars per dose (the pneumococcal conjugate vaccine is priced similarly in the United States [10], though it is covered by insurance). Although cost is likely to be barrier to pneumococcal vaccine uptake for everyone in China, it may be particularly problematic for the non-local population in China, who are sometimes known as the “floating population,” and whose presence is rapidly growing in most urban areas of the country [11].

Non-local families typically relocate from the rural agrarian areas into the more urban areas of China in search of increased economic opportunities and represent one of the largest sustained internal migrations underway globally [12]. These individuals, many of whom are economically disadvantaged, are classified as non-locals because they do not possess a local residency permit (*hukou*), which is required to access many of the important economic benefits associated with social welfare programs, including employment, education, healthcare, and social security [13–15]. Recent research has focused on determining whether disparities in access for non-locals extend to vaccination services even though the Chinese government provides EPI vaccines free of charge to all children. A study by Sun et al. (2010) examined the uptake of four vaccines on the EPI schedule by non-local populations: hepatitis B vaccine, DTP, oral poliomyelitis vaccine, and the measles vaccine. Non-locals had relatively low age-appropriate vaccination, and the authors suggested this has given rise to a pool of non-locals who are at higher risk of disease acquisition and serve as a potential reservoir for ongoing transmission of vaccine-preventable diseases in China [16]. Moreover, we have found that disparities between non-locals and locals are greater in regards to non-EPI vaccines than EPI vaccines. In a previous analysis of children <8 years of age living in Shanghai, we found that the coverage of DTP, an EPI vaccine, was similar in locals and non-locals, but there was substantially lower coverage of two non-EPI vaccines (PCV7 and a *Haemophilus influenzae* type b vaccine) among non-locals compared to locals [17].

In this study, we characterize disparities in utilization of the two pneumococcal vaccines based on residency status (local vs. non local) of children living in Shanghai. We assess whether there are differences in coverage in PCV7 and PPSV23 between local and non-local populations living in Shanghai, and examine trends in coverage of PPSV23 and PCV7 over time in children under the age of 7 years of age. We hypothesize that PCV7 has lower coverage than PPSV23 due to differentials in price, that uptake of both vaccines is lower in non-local compared to local populations, and that overage for both of these vaccines is higher among Shanghai children born more recently in the study period.

## Methods

### Data collection

A retrospective cohort was used to assess PCV7 and PPSV23 coverage by residency status in Shanghai. We used a sample of children born between 2005 and 2011 from the Shanghai Immunization Program Information System (SIPIS) housed at the Shanghai Centers for Disease Control and Prevention (CDC) in July 2012. This cohort has been described elsewhere [17]. SIPIS is an immunization information system, into which immunization providers throughout Shanghai upload pediatric vaccination administration and demographic data. Two districts, Pudong and Minhang, had their own systems at the time of data collection and are not included in SIPIS.

### Variables

For each child, SIPIS data on sex, residency (local or non-local), district of residence, birthdate, and dates of any vaccination were extracted. Urbanicity included two categories; the districts of Huangpu, Luwan, Xuhui, Zhabei, Changning, Jing’an, Putuo, Hongkou, and Yangpu are considered inner districts while the districts of Pudong, Minhang, Baoshan, Jiading, Jinshan, Songjiang, Qingpu, Nanhui, Fengxian, and Chongming are outer districts.

For both vaccines considered (PCV7 and PPSV23), a dichotomous variable was first created based on whether the child had a record of vaccine administration. Age at vaccination, the difference between vaccination date and birthdate, was calculated and categorized separately for each vaccine. This variable describes the spread of values for when the child was vaccinated, even though our analyses only included children old enough to receive the vaccine at the recommended age (i.e., 6 weeks for PCV7 and 2 years for PPSV23). For PCV7, the categories were <6 weeks, 6 weeks to <7 months, 7 months to <12 months, 12 months to <24 months, and over 24 months. Categories of less than 2 years, 2 to 3 years, and over 3 years were used for PPSV23. Co-administration of PCV7 was defined as any dose that was administered at the same time as a diphtheria-tetanus-pertussis vaccine (DTP), any measles-containing vaccine, oral polio vaccine dose 1, Hib, rotavirus vaccine, or varicella vaccine.

We calculated dose 1 coverage but not other measures (such as dose 2 uptake of PCV7 and PPSV23 or PCV7 series completion) because most children (over two-thirds) received their pneumococcal vaccinations after 2 years of age, at which point only 1 dose is recommended for both vaccines.

### Statistical analysis

Descriptive statistics including counts and proportions were calculated for each demographic characteristic and vaccination outcome. Logistic regression models were separately fit for the outcomes of PCV7 and PPSV23 administration. Unadjusted models evaluated the associations between residency, urbanicity, or birth year and vaccine administration. The multivariable, adjusted models included residency, urbanicity, and birth year together as predictors. Birth year was entered into the model as a continuous variable. For all regression models, we constructed 98 % confidence intervals and evaluated significance of findings based on an α level of 0.02. To assess trends in vaccination over time, PCV7 coverage and PPSV23 coverage were calculated by birth year and by residency, and the results depicted graphically in a bar chart. A plot of the cumulative coverage of PCV7 and PPSV23 across age of the children was stratified by residency to show trends in vaccination coverage across age. All analyses used SAS version 9.3 (SAS Institute Inc., Cary, North Carolina).

### Ethical statement

This study was deemed exempt from Institutional Review Board oversight at the University of Michigan and the Shanghai Centers for Disease Control and Prevention because it was limited to analysis of previously collected data for public health purposes.

## Results

Initially, 35,463 children were sampled from SIPIS, a minimum of 5000 from each of seven birth years examined. A total of 1822 children were removed from the dataset: 329 because of invalid vaccination dates, i.e. dates prior to their birth date, and 1493 because they did not have records of polio vaccine dose 1, DTP dose 1 or hepatitis B vaccine dose 1, 3 vaccines which have high coverage and for which the absence of records is a sign of attrition from the dataset. The resulting 33,641 children were all old enough to receive PCV7, and therefore formed the PCV7 dataset. The analysis of PPSV23 was limited to the 25,434 children older than 2 years of age and who had records of DTP dose 4 and measles-mumps-rubella vaccine, 2 EPI vaccines given before a child is 2 years of age.

Table 1 shows the study population characteristics. The total sample of 33,641 children included more males (53.6 %), greater numbers of non-locals (59.9 %), and more children from outer districts (68.9 %) than inner districts. Only 10.1 % of children in the entire study sample were vaccinated with the first dose of PCV7, and 29.8 % of children had received the first dose of PPSV23. Among those vaccinated with PCV7, most children (68.3 %) received the vaccine after 2 years of age, and the majority of children (70.3 %) who had received PPSV23 did so between 2 and 3 years of age. The mean age at vaccine administration was 2.06 years for PCV7 (standard deviation (SD): 1.07) and 2.72 years for PPSV23 (SD: 0.93).

**Table 1:** Distribution of demographic characteristics and vaccination outcomes of children, Shanghai Immunization Program Information System, July 2012

	PCV7 dataset (%)	PPSV23 dataset (%)
Overall	33,641 (100)	25,434 (100)
Sex		
Male	18,019 (53.6)	13,655 (53.7)
Female	15,622 (46.4)	11,779 (46.3)
Residency		
Local	13,496 (40.1)	11,241 (44.2)
Non-local	20,145 (59.9)	14,193 (55.8)
Urbanicity		
Inner	10,453 (31.1)	8477 (33.3)
Outer	23,188 (68.9)	16,957 (66.7)
Birth year		
2005	4753 (14.1)	4422 (17.4)
2006	4937 (14.7)	4685 (18.4)
2007	5022 (14.9)	4773 (18.8)
2008	5014 (14.9)	4724 (18.6)
2009	5030 (15.0)	4654 (18.3)
2010	4818 (14.3)	2176 (8.6)
2011	4067 (12.1)	-
Vaccinated with PCV7 Dose 1		
Yes	3395 (10.1)	-
No	30,246 (89.9)	-
Age at PCV7 Dose 1 administration^a^		
<6 weeks of age	5 (0.2)	-
6 weeks to <7 months of age	418 (12.3)	-
7 months to <12 months of age	291 (8.6)	-
12 months to <24 months of age	362 (10.7)	-
Over 24 months of age	2319 (68.3)	-
Vaccinated with PPSV23 Dose 1		
Yes	-	7583 (29.8)
No	-	17,851 (70.2)
Age at PPSV23 Dose 1 administration^b^		
<2 years of age	-	118 (1.6)
2 to <3 years of age	-	5328 (70.3)
Over 3 years of age	-	2137 (28.2)

*PCV7* pneumococcal conjugate vaccine, *PPSV23* pneumococcal polysaccharide vaccine

^a^Only includes the subpopulation of *n* = 3395 who were vaccinated for PCV7

^b^Only includes the subpopulation of *n* = 7583 who were vaccinated for PPSV23

Few children had PCV7 co-administered with another vaccine. Only 3.1 % of PCV7 dose 1 administrations occurred at the same time as another vaccine. Including all 4 doses of PCV7, only 3.5 % of these doses were given simultaneously with another vaccine.

**Fig. 1:**
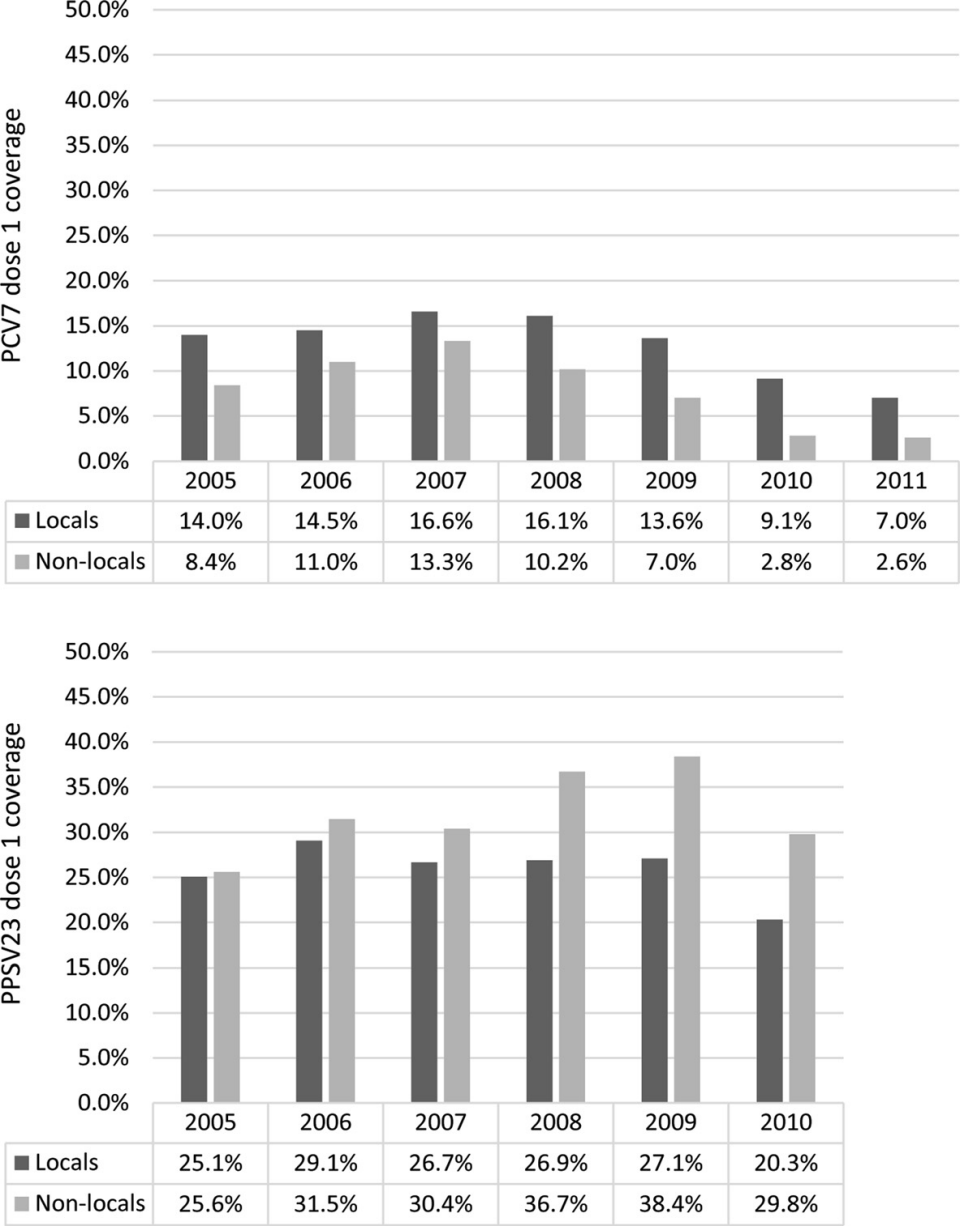
Coverage of pneumococcal conjugate vaccine (PCV7) and pneumococcal polysaccharide vaccine (PPSV23) by birth year and residency status, Shanghai Immunization Program Information System, July 2012

Vaccine coverage among the cohort decreased for PCV7 in successive years and remained stable for PPSV23 (Fig. 1). PCV7 coverage was highest for children born in 2007 (16.6 % local and 13.3 % non-local children vaccinated) whereas, only 7.0 % of local and 2.6 % of non-local children were vaccinated in 2011. For PPSV23, vaccine coverage was between 20 and 30 % for local children and between 25 and 40 % for non-local children across all years.

**Fig. 2:**
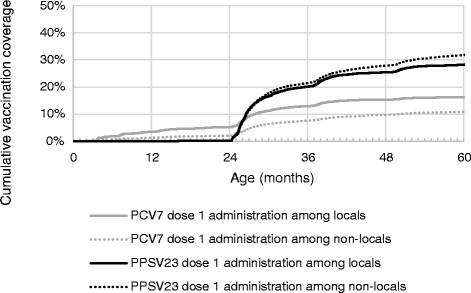
Cumulative vaccination coverage of pneumococcal conjugate vaccine (PCV7) and pneumococcal polysaccharide vaccine (PPSV23) by residency status, Shanghai Immunization Program Information System, July 2012

The plot of cumulative vaccination coverage shows that coverage of both vaccines increases substantially after 24 months of age (Fig. 2). By 90 months of age, PPSV23 coverage was higher in non-locals (37.2 %) compared to locals (30.7 %), but PCV7 coverage was higher in locals (16.4 %) than non-locals (11.1 %).

In the logistic regression, the unadjusted and adjusted models showed similar patterns (Table 2). In the adjusted model, non-locals had 0.60 times the odds of PCV7 administration compared to locals (98 % CI: 0.55, 0.66) and outer district children had 0.75 times the odds of vaccination compared to those in the inner districts (98 % CI: 0.69, 0.82). Non-locals had 1.28 times the odds of PPSV23 administration compared to locals (98 % CI: 1.19, 1.36), and those living in the outer districts had 1.23 times higher odds of vaccination than inner district children (98 % CI: 1.15, 1.32).

**Table 2:** Odds ratios (OR) and 98 % confidence intervals (CI) for administration of the first dose of pneumococcal conjugate vaccine (PCV7) or pneumococcal polysaccharide vaccine (PPSV23) in children; Shanghai Immunization Program Information System, July 2012

	PCV7 administration		PPSV23 administration	
	Unadjusted model OR (98 % CI)	Adjusted model OR (98 % CI)	Unadjusted model OR (98 % CI)	Adjusted model OR (98 % CI)
Non-local vs local	0.57 (0.52, 0.62)	0.60 (0.55, 0.66)	1.33 (1.25, 1.42)	1.28 (1.19, 1.36)
Outer vs inner district	0.67 (0.61, 0.73)	0.75 (0.69, 0.82)	1.30 (1.21, 1.40)	1.23 (1.15, 1.32)
Birth year (continuous)	0.85 (0.83, 0.87)	0.86 (0.84, 0.88)	1.05 (1.03, 1.07)	1.04 (1.02, 1.06)

## Discussion

In a large sample of children from Shanghai, China, we found uniformly low pneumococcal vaccine coverage for both the PCV7 and PPSV23 for all birth years; this was especially true for PCV7 administration in non-locals and in younger infants. Although Shanghai is one of the wealthiest subdivisions of China, its PCV7 coverage levels are similar to those from a 2012 survey of children aged 1 to 2 years selected from 31 provinces throughout China in which just 9.91 % had received a dose of PCV [18]. These figures are in stark contrast to pneumococcal vaccine coverage in many countries where these vaccines are included on the EPI schedule; the 2009 US National Immunization Survey put PCV7 coverage among children aged 19–35 months at 92.6 % for ≥3 doses and 80.4 % for ≥4 doses [19]. And, in developing countries funded through the GAVI Alliance, 19 % of children received a third dose of PCV [20].

These estimates of pneumococcal vaccine coverage in China are far below those estimated for coverage with EPI vaccines. For example, 98.6 % of children in Tianjin in 2012 had received measles vaccine dose 1 [21], and over 97 % of children in Shanghai in 2012 had received DTP dose 1 [17]. The discrepancy in coverage is likely due, in part, to the pneumococcal vaccines’ lack of inclusion on China EPI schedule and the need to pay for them out of pocket. In contrast, EPI vaccines are mandatory and free to the public. Vaccine cost appears to be especially important given the higher coverage but lower cost of PPSV23 compared to PCV7. The $100 price difference between these two pneumococcal vaccines could potentially incentivize caregivers to purchase the less expensive, yet less effective, PPSV23. Nonetheless, China has a significant number of childhood IPD cases, second only to India globally [2], and the high disease burden, particularly among young infants who are also most likely to experience severe illness and death, is unlikely to change without higher vaccination coverage against PCV7 or PPSV23.

We found pneumococcal vaccine coverage differences by residency status. The local population had a significantly higher PCV7 dose 1 coverage than the non-local population, although contrary to our initial hypothesis, non-locals had a higher PPSV23 dose 1 coverage than local populations. Because locals generally have higher household incomes than non-locals [14], they may be more able to afford the higher cost of the PCV7 vaccine while non-locals may opt instead for the less expensive PPSV23 alternative. As a non-conjugated polysaccharide vaccine, PPSV23 has lower efficacy than for PCV7 (>90 % for PCV7 vs 60–70 % for PPSV23) [3, 4], and lower coverage with PCV7 may result in non-local children remaining more vulnerable to pneumococcal infection, even with PPSV23 vaccination. For children born later in the study, in 2009 and 2010, coverage with any pneumococcal vaccination was higher for non-locals than locals because of substantially higher coverage of PPSV23. We do not have information on why non-locals, characterized but economic disadvantage vis-à-vis locals, had higher PPSV23 coverage in these years but it could result from non-locals having greater worry about disease and less concern about side effects than locals.

As a conjugated vaccine, PCV7 can be administered at younger ages compared to PPSV23, although we found most children were vaccinated against pneumococcus with either vaccine when over 2 years of age. The PCV7 was only approved for use in China after 2008, and earlier birth cohorts would have been unable to receive this vaccine at younger ages. There was a decrease in PCV7 coverage for later birth years, perhaps because parents became accustomed to vaccinating their children later, and children in later birth cohorts had not yet reached 2 years of age. Vaccination at earlier ages is preferred as it provides protection against more serious disease in young infants, but that also necessitates more doses of PCV7 for series completion and long-lasting immunity. It may be that parents object to earlier vaccination on financial grounds despite the risk of more serious disease for younger children or it could be that vaccinating at an earlier age is not an option because the manufacturer’s instructions for PCV7 recommend against co-administered with any other vaccine, although there is no such contraindication in the United States or Europe. Moreover, the EPI schedule for children under 1 year of age in China, like many other countries, is complex and involves many vaccinations and parents may push back against additional shots for their children.

The choice of promoting PCV7 or PPSV23 is an important one, because healthy children are not recommended to be immunized with both vaccines. Because of the inherent immunological limitations to PPSV23, it is not recommended for routine childhood use in the United States [22], and international pneumococcal control has rather focused on conjugate vaccines [7]. Therefore, we would encourage adoption of PCV7 over PPSV23, although data on the distribution of pneumococcal serotypes among IPD cases in China (which are currently lacking [23]) could mitigate this recommendation if proportionally few cases result from the serotypes in the 7-valent vaccine.

This study has several notable limitations and strengths. The database of SIPIS lacks data for Pudong and Minhang, two populous districts in Shanghai which may reduce the representativeness of the data and bias overall coverage estimates. Because SIPIS was only implemented after 2010, information for children in prior years may be limited. This database only includes children who receive services in immunization clinics and others would not be present in the system, which may result in vaccination coverage overestimation, particularly for non-locals who probably are less likely to access these services than locals. Attrition can also occur in SIPIS; families who move out of Shanghai are not necessarily removed from the system. We tried to limit this bias by creating a dataset which only includes individuals who received sentinel vaccines. Lastly, this study had a narrow time frame and because PCV7 was introduced in 2008 in Shanghai, we were unable to evaluate longer-term trends in uptake. A follow-up study would permit assessment of trends over time, particularly between locals and non-locals and in the age at administration of pneumococcal conjugate vaccine dose 1. The strengths of this study included data involving a large number of children were obtained from a well-established immunization information system, and so we were able to obtain accurate information on dates of vaccination over a number of years.

## Conclusions

We found low vaccination levels with both pneumococcal vaccines among children in Shanghai, China over a 7-year span of time. The low coverage could help explain the continuing high burden of childhood pneumococcal disease observed throughout China if similar patterns in vaccine coverage characterize the remainder of the country. Although PPSV23 coverage was comparable or higher in non-locals compared to locals, PCV7 coverage was uniformly lower among non-local populations for all years, potentially placing them at higher risk of disease due to the lower efficacy of the polysaccharide vaccine. Age at initial vaccination was above that recommended for both PCV7 and PPSV23, leaving young infants susceptible to disease at a time that they are especially vulnerable to more severe illness and death. Shanghai should consider the addition of PCV7 to the EPI schedule to increase coverage. However, any targeted immunization program aimed at preventing pneumococcal disease should ensure equitable access to vaccines for non-locals and locals alike and needs to be provided at an early age so that the burden of invasive pneumococcal disease is reduced among the very young.

## Abbreviations

CDC: Centers for Disease Control and Prevention
DTP: diphtheria-tetanus-pertussis vaccine
EPI: expanded program on immunization
PCV7: 7-valent pneumococcal conjugate vaccine
PPSV23: 23-valent pneumococcal polysaccharide vaccine
